# Science diplomacy: A global research field? Findings from a bibliometric analysis of the science diplomacy scholarship of the past twenty years

**DOI:** 10.1007/s11192-025-05396-x

**Published:** 2025-08-08

**Authors:** Anna-Lena Rüland, Lise H. Andersen, Alan Kai Hassen, Carringtone Kinyanjui, Annika Ralfs, Bruno Iochins Grisci

**Affiliations:** 1 University College London, London, UK; 2 Leiden University, Leiden, The Netherlands; 3 University of Manchester, Manchester, UK; 4 Lund University, Lund, Sweden; 5 Federal University of Rio Grande Do Sul, Porto Alegre, Brazil

**Keywords:** Science diplomacy, Bibliometrics, Internationalization, Large language models, Network analysis

## Abstract

Science diplomacy is a unique research field that is driven and shaped by scholars and practitioners alike. This study examines whether and how recent trends in the broader science diplomacy discourse have impacted scholarship on the topic. First, it examines whether the pertinent scholarship is as international in outlook as practitioners have made science diplomacy out to be. Second, the study investigates whether recent calls to diversify the science diplomacy scholarship have gained traction. It does so by examining how diverse the science diplomacy scholarship is in terms of: (i) The geographical distribution of authors, (ii) the geographical distribution of funding sources as well as (iii) the geographical area that is being studied in science diplomacy publications. Using a network analysis and a large language model-enhanced bibliometric analysis, the study shows that the internationalization of the field—both in terms of author affiliations and geographical area being studied in publications—is only slowly advancing and is currently restricted to a few regions, with the United States and Europe clearly dominating the production of knowledge on science diplomacy. Overall, the study’s findings thus corroborate past claims that the science diplomacy scholarship exhibits North–South dynamics similar to those in other research fields.

## Introduction

Scholars have long been interested in the intersection of science and international relations (e.g. Skolnikoff, 1967), presumably because of the myriad ways in which science affects international affairs and vice versa (Weiss, 2015). Only in the early 2000s, however, did policymakers and scholars start to label activities at this intersection as “science diplomacy”. Since then, the interest in science diplomacy as a strategic tool in a globalized, knowledge-based economy has increased steadily, both in the social (e.g. Kaltofen & Acuto, 2018; Smith III, 2014; Yen, 2024) and natural sciences (e.g. Bezak et al., 2023; Hennessey, 2019).

What distinguishes science diplomacy as a research subject and field from others is that it is driven by practitioners (i.e. policymakers and scientists-turned-policymakers) and scholars alike. Since the emergence of the science diplomacy concept in the early 2000s, both actor groups have shaped the broader discourse on the topic. Between the early 2000s and 2015, practitioners dominated the discourse on science diplomacy. During this phase, science diplomacy was largely understood and framed as a practice that is firmly embedded in and shaped by the liberal international order, with practitioners highlighting international collaboration (Lord & Turekian, 2007) and scientific internationalism as being key to address global challenges (Fedoroff, 2009). Only after 2015 did a more nuanced discourse on science diplomacy emerge. This discourse, largely initiated through several seminal scholarly studies on science diplomacy, highlighted the “dual logic” of the practice. According to this logic, science diplomacy can be used both for collaborative and competitive ends, meaning to advance national interests and to address global challenges (Ruffini, 2020). In the past few years, the science diplomacy discourse has changed yet again. On the one hand, several scholars have underlined a lack of diversity in and the colonial legacy of science diplomacy (Andersen, 2018; Polejack et al., 2022). On the other hand, since Russia’s invasion of Ukraine in 2022, both practitioners and scholars have increasingly argued that science diplomacy has entered a “post-naive” era (Olšáková & Robinson, 2022; The Royal Society & American Association for the Advancement of Science, 2025), which is characterized by the securitization of national science systems and international scientific exchanges.

This study investigates whether and how the broader science diplomacy discourse has shaped the science diplomacy scholarship. It does so in two ways. First, against the backdrop of the practitioner-driven framing of science diplomacy as an inherently international endeavor, it examines how international science diplomacy scholarship is. Second, the study investigates whether recent calls to diversify science diplomacy scholarship have been successful. The study does so by examining how diverse the science diplomacy scholarship of the past two decades is in terms of: (i) The geographical distribution of authors, (ii) the geographical distribution of funding sources as well as (iii) the geographical area that is being examined in science diplomacy publications. To do so, the study uses a network analysis and a large language model (LLM)-enhanced bibliometric analysis, an approach that has rarely been tried and tested in the context of bibliometric studies (for exceptions see: Fijačko et al., 2024; Xu et al., 2024). Adding manual evaluation and refinement checks to the LLM-enhanced data collection process, the study demonstrates that LLMs like OpenAI’s GPT-4 Turbo Preview (GPT-4) (OpenAI et al., 2024) perform well in identifying relevant abstracts, even when the criteria are complex.

Using the described LLM-enhanced bibliometric analysis, this study addresses the following questions: (i) Which countries and regions have contributed to the science diplomacy scholarship?; (ii) which countries and regions have funded research on science diplomacy?; and (iii) which countries and regions have science diplomacy scholars studied? In doing so, the study provides an overview of the evolution of the still nascent, but quickly expanding, scholarship on science diplomacy along geographical criteria. As science diplomacy research is regularly used to inform and justify decisions in science and innovation policy that directly affect researchers (Ledgerwood & Bunn, 2022), it is crucial for the global science community and policymakers to understand whose voices are well-represented in the scholarship and whose are not. By providing an overview of the geographical patterns in the scholarship on science diplomacy, this study also offers insights into the dynamics of national versus international engagement as well as North–South and South–South relations within the science diplomacy scholarship. In this study, North–South terminology is used to refer to a “history of colonialism, neo-imperialism, and differential economic and social change through which large inequalities in living standards, life expectancy, and access to resources are maintained” between high-income as well as middle- and low-income countries (Dados & Connell, 2012). Studying North–South dynamics is not only crucial from an equity in science perspective, but also vital because science diplomacy has been argued to be a means through which a nation or region can advance its interests (see: Flink & Schreiterer, 2010), providing those who have the resources and knowledge to engage in this practice with a political and economic advantage.

Overall, the study’s results indicate that despite the practitioner-driven framing of science diplomacy as an inherently international endeavor, there is a pronounced national focus in the science diplomacy scholarship, both in terms of outlook and collaboration patterns. For instance, all central contributors to the science diplomacy scholarship study their own national science diplomacy system extensively and most publications are written by authors based in the same country, with a majority of these being single-authored contributions. This latter pattern is unsurprising given that humanities and social science scholars have so far contributed considerably to the science diplomacy scholarship.

In addition, the study indicates that recent calls to diversify the science diplomacy scholarship have had limited success so far. The internationalization of the field, both in terms of author affiliations and geographical area being studied in publications, is only slowly advancing. Indeed, the study’s results corroborate past claims (Polejack et al., 2022) that the science diplomacy scholarship exhibits North–South dynamics similar to those in other research fields (e.g. Bai, 2018). For instance, a majority of the literature concentrates on Europe and the United States (US). Moreover, both the early and more recent science diplomacy scholarship has predominantly come from the US and Europe. Europe—both as a region as well as through the European Union (EU)—is also the most prominent funder of science diplomacy research. Only in the last few years have scholarly contributions from authors affiliated with research institutions in Latin America and Asia started to increase, with scholars based in Oceania and Africa having contributed the least to the literature. This is unsurprising given that with the exception of a recent surge in funding from Asian institutions, Africa and Oceania are underrepresented as regional funders of science diplomacy research.

These findings highlight the continued need for more equitable research collaborations and practices in the science diplomacy field. On the part of research funders, this study shows that international research funding schemes and exchange platforms that facilitate interactions and collaborations between researchers based in the Global North and global South, are currently lacking. On the part of the research community, the study demonstrates that personal reflexivity (Polejack et al., 2022) and collaborations on a smaller scale can positively impact the diversity of science diplomacy scholarship.

The remainder of this article is structured as follows: Section two provides a qualitative review of the major themes that have dominated the science diplomacy literature of the past two decades. Thereafter, section three outlines the study’s research design, while section four presents the main findings of the bibliometric analysis. Section five then critically discusses the study’s findings and their implications for policy making. Finally, section six outlines the study’s limitations, and indicates future avenues for research on science diplomacy.

## Literature review

Although the concept of science diplomacy has gained considerable traction across different disciplines in recent years, the scholarship on the topic has yet to be systematically mapped. The present study depicts the first attempt to contribute a comprehensive bibliometric overview of the science diplomacy scholarship, mainly along geographical criteria. This interest in geographical patterns is informed by past conceptualizations of science diplomacy.

### Defining science diplomacy

Much of the existing science diplomacy scholarship traces the concept’s origins back to a meeting that the British Royal Society (RS) and the American Association for the Advancement of Science (AAAS) organized in 2009. At this meeting, different stakeholders, including diplomats and scientists, came together to discuss “how science can contribute to foreign policy objectives” (The Royal Society & American Association for the Advancement of Science, 2010, p. v). The discussions held at the meeting inspired a report that conceptualizes science diplomacy as:

- Science in diplomacy (scientific evidence informing foreign policy);
- Diplomacy for science (diplomacy facilitating international science cooperation), and
- Science for diplomacy (using international science cooperation to improve international relations).

To substantiate this conceptualization, the RS-AAAS report also provides examples of each science diplomacy dimension. For instance, the Intergovernmental Panel on Climate Change is seen to be a prime example of scientific advice informing international environmental policy. The European Organization for Nuclear Research, in turn, is cited as an example of how diplomatic actors can facilitate cutting-edge science cooperation across national boundaries and how such cooperation can open channels of communication between historically estranged political communities like Germany and Israel (The Royal Society & American Association for the Advancement of Science, 2010, pp. 9–12).

While practical and catchy, the widely circulated RS-AAAS taxonomy of science diplomacy has not remained unchallenged and static over time. For instance, two recent reports on science diplomacy -one published by the European Commission; the other by the RS and AAAS-argue that the RS-AAAS science diplomacy taxonomy needs to be adapted to the current geopolitical context (Gjedssø Bertelsen et al., 2025; The Royal Society & American Association for the Advancement of Science, 2025). Both reports specifically highlight the competitive and realpolitik dimensions of science diplomacy. At the same time, they stop short of completely overhauling the original RS-AAAS taxonomy, as the European Framework on Science Diplomacy merely adds a fourth dimension to the original typology (diplomacy in science) and the 2025 RS-AAAS report on science diplomacy simplifies the original taxonomy by merging the three core dimensions of science diplomacy into two (science impacting diplomacy and diplomacy impacting science).

Several alternative definitions of science diplomacy that were formulated prior to 2025 underline the different geographical levels that science diplomacy initiatives target. For example, Peter Gluckman, former science advisor to New Zealand’s prime minister, has proposed a more “pragmatic” definition of science diplomacy as actions designed to:

- Directly advance a country’s national needs, such as attracting top researchers from abroad;
- Address cross-border interests like river pollution, and/or
- Meet global needs and challenges, such as climate change (Gluckman et al., 2017).

Building on the RS-AAAS and Gluckman et al.’s science diplomacy conceptualizations as well as critiques that these conceptualizations have elicited, Rüffin and Rüland (2022) equally underline that science diplomacy has three distinct levels of engagement, namely the national, regional, and global.

### Science diplomacy at the national, regional, and global level

In line with what several alternative science diplomacy conceptualizations indicate, a more in-depth, qualitative analysis of the scholarship on science diplomacy reveals that much of it has an explicit or implicit geographical focus. Firstly, science diplomacy scholarship has thoroughly studied national approaches to the practice. This is not surprising, given that science, technology, and innovation are typically seen to be a cornerstone of a nation state’s (knowledge) economy (Wagner, 2002). Accordingly, science diplomacy scholars have analyzed the main science diplomacy actors, institutions, and strategies of different states. A majority have investigated national science diplomacy approaches of European and North American states, including, but not limited to, France (Lane, 2013), Germany (Epping, 2020), and Canada (Sabzalieva et al., 2021). However, there are also some analyses of national science diplomacy approaches in emerging economies like South Africa (Masters, 2016), China (Su & Mayer, 2018), and Russia (Kharitonova & Prokhorenko, 2020).

In addition, there is a large share of science diplomacy studies that have investigated the role of science diplomacy in governing certain world regions. For instance, in 2011, Berkman et al. dedicated an entire edited volume to the issue of “Science Diplomacy: Antarctica, Science and the Governance of International Spaces” (Berkman et al., 2011). Among other things, the chapters of the volume delve into the role of science and diplomacy in developing the Antarctic Treaty System, sustainably managing the Southern Ocean, and tackling the Antarctic Ozone Hole. Studies that examine the role of science diplomacy in governing the Arctic and the Atlantic are similarly common (e.g. Polejack, 2021; Polejack et al., 2021).

As Flink and Rüffin (2019) have indicated in their non-systematic review of the science diplomacy scholarship, science diplomacy studies also often pay particular attention to the role of science (diplomacy) in addressing grand challenges, which are global in nature. Özkaragöz Doğan et al. (2021), for instance, examine how South-South and North–South collaborations in science and technology can help inform climate change mitigation and adaptation strategies. Hornsby and Parshotam (2018), in turn, have investigated how science diplomacy can be used to increase the participation of Sub-Saharan African countries in international food standard setting negotiations. Another example of this line of research are a set of studies that appeared following the outbreak of the Covid-19 pandemic in 2020 and explore policy responses to the pandemic from a science diplomacy perspective (e.g. Echeverria et al., 2020; Mencía-Ripley et al., 2021).

In sum, the pertinent literature thus highlights the relevance of science diplomacy at different geographical levels. On the national level, countries benefit from defining their needs and interests as well as ways of advancing these through science. Beyond that, science diplomacy is employed to address challenges of regional and global scope for which international collaboration appears immensely beneficial, if not compulsory.

### The Global South in science diplomacy

In recent years, several initiatives to “globalize” the scholarship on science diplomacy have emerged (Barrett & Homei, 2024; Büyüktanir Karacan & Ruffini, 2023). Most of these initiatives emphasize that there is a need for science diplomacy studies of and from the global South because this particular world region faces different challenges and sets different priorities for science policy than the Global North. The latter predominantly engages in science diplomacy to further advance access to researchers and research findings, to extend its political influence, and to promote national achievements in research and development (Flink & Schreiterer, 2010, p. 669). In contrast, in emerging economies, “the debate about science diplomacy has inevitably tilted towards developmental objectives” (Polejack et al., 2022, pp. 11–12). Büyüktanir Karacan and Ruffini (2023, p. 744), who have assembled a special issue on “Science Diplomacy in the global South”, likewise underline that “the primary focus of science diplomacy in the South is on national interests and needs, with the priority objective” of development and capacity-building. Contributions to Karacan’s and Ruffini’s special issue seem to support this claim. Rüland et al. (2023), for instance, argue that emerging economies of the global South typically join Big Science collaborations to strengthen their national science and technology capacities. Robinson et al., (2023, p. 750) similarly contend that the 1970s saw a globalization of science diplomacy activities because “the increasingly numerous and diverse countries around the world were clamoring for a more equitable distribution of scientific expertise and technological capacity”.

This study adds to the existing scholarship on science diplomacy by systematically exploring (i) how global, as opposed to national, the scholarship is in terms of authorship, funding, and outlook as well as (ii) investigating to what extent recent initiatives to globalize the field are reflected in these three dimensions.

## Methods and data

The study’s empirical backbone forms an LLM-enhanced bibliometric analysis. The following section first outlines how the bibliometric data was extracted, cleaned, and curated and thereafter describes how the bibliometric data was analyzed and visualized.

### Data extraction, collection, and curation

To capture the broadest possible scope of scholarly contributions on the topic of science diplomacy, all peer-reviewed publications whose abstract contained one of the search terms depicted in Table 1 were bulk-extracted from Scopus in November 2023. The search terms were chosen by three domain experts that are familiar with the science diplomacy scholarship and the terms that are commonly used to refer to activities at the intersection of international affairs and science. The extraction was done via the Scopus Application Programming Interface using the “PyScopus Scraper” (Zuo et al., 2017) and yielded a sample of 811 publications. The scraped bibliometric metadata was recorded in an Excel spreadsheet and included, among others, each publication’s title, abstract, year of publication, digital object identifier, funding source and the authors’ institutional affiliation (see supplementary material for more information; link to dataset on publicly accessible repository: https://github.com/BrunoGrisci/science-diplomacy-global-research).

**Table 1:** Overview of used search terms

Search terms
Science diplomacy
Scientific diplomacy
Science and technology diplomacy
Science and innovation diplomacy

As is widely acknowledged in the literature, using Scopus as a bibliometric database comes with several limitations (Asubiaro et al., 2024). For example, the rigorous assessment and selection processes of Scopus tend to exclude local, national, and regionally focused publications. This may lead to an underrepresentation of research from regions other than North America and Europe as well as of articles that are published in languages other than English (Asubiaro et al., 2024, p. 1470). Yet, in comparison to other databases, Scopus also has several advantages. For instance, Scopus covers a broader range of journals in the humanities and social sciences (Mongeon & Paul-Hus, 2016) and has a wider geographical scope than, for example, the Web of Science (Chinchilla‐Rodríguez et al., 2012). Given that this study’s objective was to map the global science diplomacy scholarship, which the social sciences and the humanities have considerably contributed to, these two characteristics were considered important. Scopus was also considered an appropriate choice because a cross check of alternative bibliometric databases (like Scielo and CNKI) with the search terms translated into Spanish, Portuguese, and Chinese showed that science diplomacy-related publications are not as broadly covered in these regional databases as they are in Scopus.

**Fig. 1:**
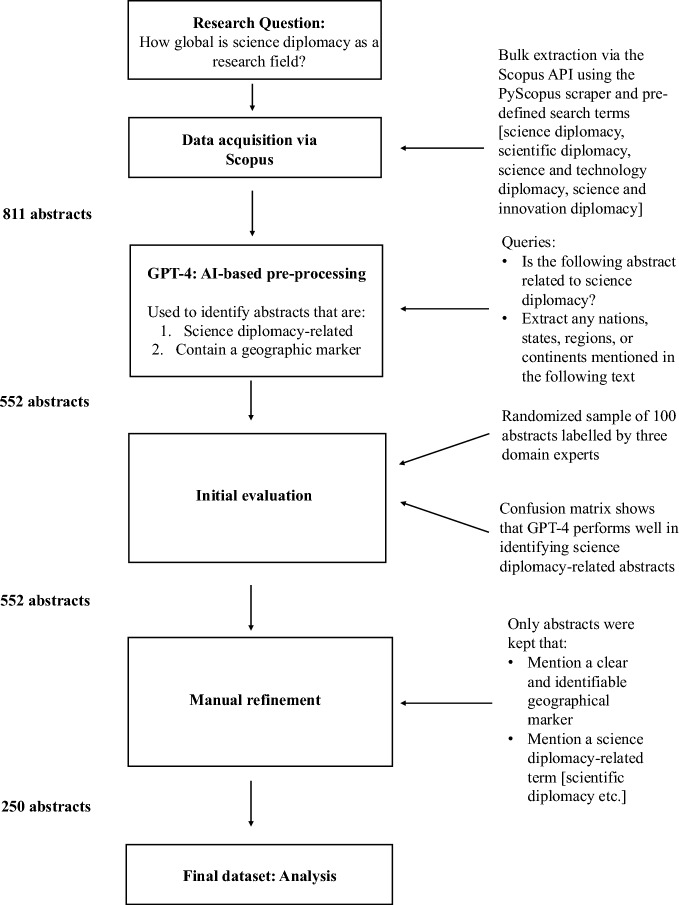
Overview of data collection process

Following the bulk extraction, the dataset was refined in three steps (see Fig. 1). First, OpenAI’s LLM GPT-4 was initially evaluated on the dataset to see whether it generally managed to identify abstracts that are science diplomacy-related and contain a geographical marker. Mirroring these two selection criteria (science diplomacy-related or geographical marker), the queries that were used for GPT-4 read as follows: (i) “Is the following abstract related to science diplomacy?”; (ii) “Extract any nations, states, regions, or continents mentioned in the following text”. Next, to benchmark how well GPT-4 performs in selecting relevant abstracts, a randomly selected subset of 100 GPT-4 evaluated abstracts was manually labeled by three domain experts as an initial evaluation dataset. On this initial evaluation dataset GPT-4 performed almost as well in identifying science diplomacy-related abstracts as the domain experts did (see confusion matrix in the appendix). The model labeled 86 percent of the subset correctly (accuracy), while only missing four science diplomacy-related articles (false negatives). Because of its high accuracy in identifying relevant abstracts, the model was used for the entire dataset. In total, GPT-4 excluded 259 abstracts based on the selection criteria as not science diplomacy-related, leaving 552 abstracts in the dataset. Third, as a quality assurance, these 552 abstracts were manually checked for relevance and to identify potential GPT-4 mistakes. Before this round of manual labeling, several conceptual decisions were made. To avoid conceptual blurring, it was decided that abstracts which touch on related but distinct concepts from science diplomacy (e.g. public diplomacy, cultural diplomacy) would not be included in the dataset (Table 2 provides an overview of terms and concepts that signal whether an abstract was included in the dataset or not). This also applies to concepts such as technology diplomacy, space diplomacy, knowledge diplomacy, energy diplomacy and international scientific cooperation that are often equated with science diplomacy. The underlying reasoning for this decision was that all of these concepts are distinct from science diplomacy in that:

- The main focus is on the applied side of science (energy, space and technology diplomacy);
- The outcome is seen to be a win–win for everyone involved (knowledge diplomacy); and
- The activity lacks a distinct political dimension (international scientific cooperation).

**Table 2:** Overview of terms and concepts used during data collection

Guiding terms and concepts for data selection
Science diplomacy
Scientific diplomacy
Ocean science diplomacy
Science and technology diplomacy
Science, technology and innovation diplomacy
Science and innovation diplomacy
Polar science diplomacy

Given the study’s interest in geographical patterns in the science diplomacy scholarship, it was decided that only abstracts that mention geographical markers like cities, countries, subregions, or regions would be kept in the database. At the end of this manual refinement process, the number of abstracts in the dataset was reduced to 250.

In the following step, geographical markers mentioned in the abstracts were manually classified and aggregated according to the United Nations (UN) Geoscheme (United Nations, 2024). This scheme categorizes geographical areas into countries, sub-regions, and regions. For instance, if an abstract mentioned “Japan” or a subnational area that was known to be part of Japan, this country-level information was noted down explicitly and classified as talking about “Eastern Asia” at the sub-regional level. At the regional level, the same article was categorized as having a focus on “Asia”. This approach allowed the study to analyze data at national, regional, and global levels; however, it is important to note that it was only possible to aggregate from lower to higher levels. For example, where a sub-region or region was mentioned in an abstract, it was impossible to extrapolate this information to the country level. The decision to aggregate geographical markers also required several other judgement calls. For instance, where abstracts used historical country denominations (e.g. German Democratic Republic), contemporary names (e.g. Germany) were used instead per the UN Geoscheme. In cases where abstracts grouped countries together under common abbreviations like “BRICS”, the respective countries that belonged to the grouping in 2024 were noted individually. Broad, contested, and ambiguous geographical markers like “global South”, “developing countries” or “the West” were labelled as “none”. Moreover, during the data collection process, it was noted that certain geographical areas (e.g. the Atlantic, Arctic, and Antarctica) featured extensively in the dataset, but were not, or only partially, covered in the UN Geoscheme. To capture this regional focus in the science diplomacy literature, these geographical areas were nonetheless included in the dataset.

To classify and aggregate author affiliations, the same procedure as described above for geographical markers mentioned in abstracts was used. With the exception of a few cases (n = 2), affiliation information was available in Scopus and thus, it was rarely necessary to use the label “none”.

Finally, to analyze funding patterns in the science diplomacy scholarship, funding information that was publicly available, was manually added where it was missing in the bulk-extracted data. Overall, this information was available for roughly half the dataset. Like the author and geographical marker information, the funding information was classified and aggregated according to the UN Geoscheme. Funders like the UN or World Health Organization were labeled as multilateral organizations. Because of its prominence as a funder, the EU was kept as a separate funder category.

### Data analysis and visualization

The data were analyzed and visualized using descriptive statistics and network analysis. Stacked bar charts visualize trends with regards to author affiliations as well as collaboration patterns across country and regional levels. Stacked bar charts are also used to visualize which regions are mentioned in abstracts over time. In the following, regions and countries that are studied in science diplomacy publications are referred to as “targets” and visualizations that are associated with this unit of analysis have been labeled accordingly (e.g. target network). Funding patterns in the science diplomacy scholarship over time are visualized via a line graph.

To uncover patterns of relationships in the science diplomacy scholarship, the data was analyzed using network analysis. Centrality measures were calculated for two types of networks: (i) The collaboration network (i.e. who collaborates with whom on a publication) and (ii) the target network (i.e. who mentions who in an abstract). Calculating centrality measures is important because they provide insights into how pivotal each country or region is in the scholarly discourse (Bloch et al., 2023). Degree centrality is calculated as the number of direct connections (edges) a node has (Borgatti & Brass, 2019). For directed networks, such as the target network in this study, it can be separated into in-degree (incoming edges) and out-degree (outgoing edges) centrality. In the case of the target network, degree centrality identifies regions with the most direct mentions. These are the most frequently mentioned or mentioning regions, indicating their immediate prominence in the science diplomacy literature. In-degree centrality identifies regions most frequently mentioned in the science diplomacy literature. Out-degree centrality identifies regions that are highly active in mentioning others, potentially acting as knowledge disseminators or contributors to the field.

Betweenness centrality measures the extent to which a node acts as a bridge along the shortest paths between other nodes (Borgatti & Brass, 2019). It highlights regions that serve as crucial intermediaries, facilitating connections between otherwise disconnected regions in the scholarship. These are strategic nodes for bridging disparate areas of discourse. Closeness centrality reflects how close a node is to all other nodes in the network (Borgatti & Brass, 2019). It is calculated as the reciprocal of the sum of the shortest path distances from a node to all other nodes. A region with high closeness centrality is well-connected to the network, meaning it can access information from other regions with minimal steps. This indicates a region’s central position in the scholarship.

Eigenvector centrality assigns importance to nodes based on their connections to other important nodes (Borgatti & Brass, 2019). It is derived from the principal eigenvector of the adjacency matrix of the network and emphasizes regions that are not just well-connected in general but connected to other influential regions.

The collaboration and target networks were created for the period 2002–2023. Moreover, to uncover trends over time, a degree centrality ranking chart was created for the collaboration network (see Fig. 7). The chart covers four distinct time brackets, namely 2002–2008, 2009–2012, 2013–2019, and 2020–2023. These time brackets were either chosen for pragmatic reasons or because they depict important phases in the development of the science diplomacy scholarship. For instance, 2009 was chosen as the starting point of the second time bracket because the RS-AAAS meeting that led to the publication of the seminal report on “new frontiers in science diplomacy” took place that year. To explore whether any relevant research on science diplomacy had been published before 2009, it was decided to cover the years leading up to the publication of the report in another time bracket. Moreover, as the first special issue on science diplomacy in Latin America was announced around 2020, it was determined that the last time bracket should cover the period between then and 2023, the last year for which data was available. Finally, to keep the time intervals somewhat balanced, it was decided to include a fourth time bracket for the period 2013–2019.

## Results

**Fig. 2:**
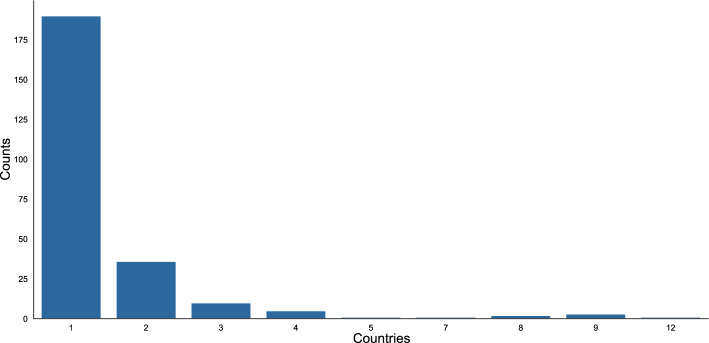
Number of author affiliations per publication

As Fig. 2 illustrates, the majority of publications in the dataset are written by authors based in the same country. These publications (n = 188) represent both single-authored papers (n = 118) (see Fig. 11 in appendix) and national collaborations (n = 70). Publications that are written by authors distributed across two or more countries (n = 125)—in the following referred to as international publications—are clearly in the minority.

**Fig. 3:**
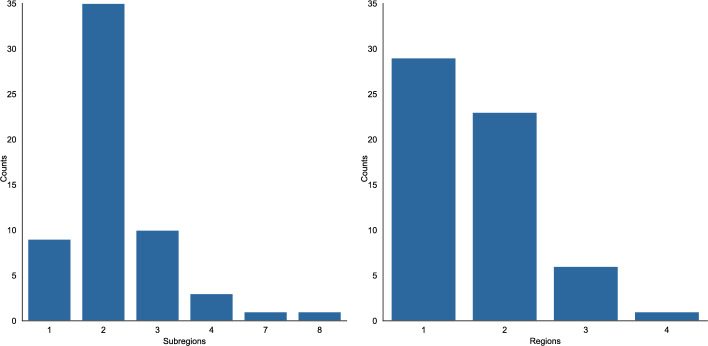
Number of author affiliations at the subregional and regional level per international collaboration

Among international publications, the majority of articles are written by authors affiliated with two distinct subregions (see Fig. 3). Evaluating the number of regional affiliations, most internationally collaborating authors are based in the same region. However, collaboration across two regions is almost as common as that within the same region (see Fig. 3). Hence, while national publications are most common, when researchers publish internationally, their collaboration frequently spans two subregions, either within the same region or across two regions.

**Fig. 4:**
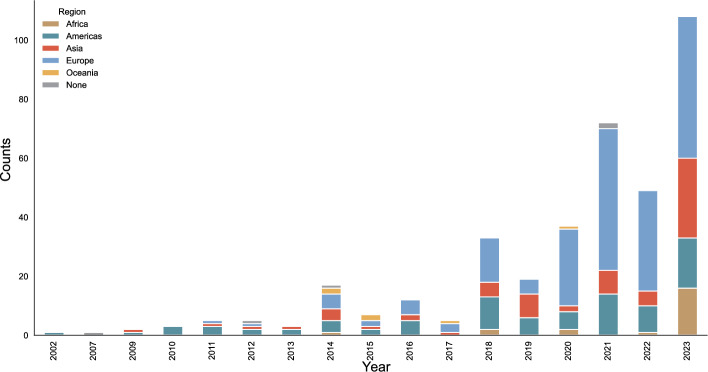
Author affiliation aggregated at the regional level over time

Considering author affiliation since the early 2000s (see Fig. 4), it is possible to discern a gradual trend towards internationalization. Authors associated with the Americas have been consistently contributing to the literature since 2002. While they contributed a relevant share of science diplomacy-related publications since at least the early 2010s, the vast majority of contributions published on the topic since 2020 have been written by scholars affiliated with Europe. Only in 2023 did publications to the literature by authors with African and Asian affiliations become more prominent, experiencing a marked increase from previous years. Out of the five regions, authors affiliated with Oceania feature the least.

**Fig. 5:**
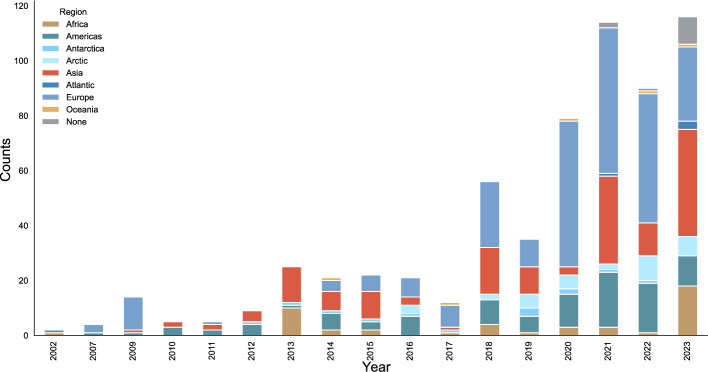
Regions mentioned in abstracts over time

When exploring target regions mentioned in article abstracts over time (see Fig. 5), there has also been a trend towards slow diversification. The Americas have been consistently mentioned in abstracts every year (except for 2017), experiencing a rather steady annual increase in attention. For Europe, a drastic increase of mentions can be observed especially from 2018 onwards. Considerations of Asia have gained a greater presence in the literature – particularly in publications released in 2018, 2021, and 2023. In 2013 and 2023, mentions of Africa were the largest in the period under examination. Furthermore, in more recent years (2018–2023), mentions of the Arctic have had a steady presence, and in 2023, the biggest share of abstracts that did not indicate a clearly identifiable world region were published.

**Fig. 6:**
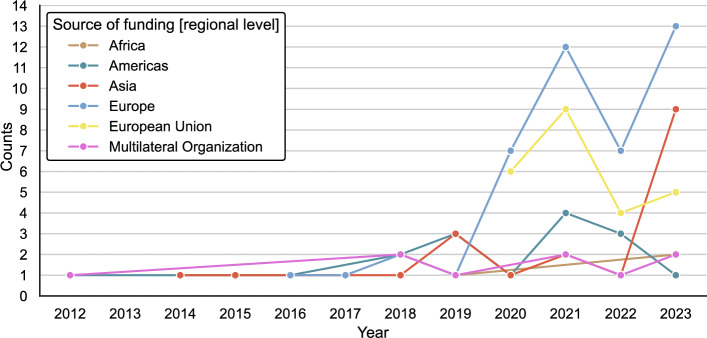
Funders of science diplomacy research over time (aggregated at the regional level)

Considering funders of science diplomacy research over time, aggregated at the regional level, European countries have been the biggest investor (independent of EU initiatives) (see Fig. 6). While the EU was the second biggest funder of science diplomacy research in the period 2020–2022, it was overtaken by Asian sponsors, whose funding has increased substantially from 2022–2023. Funding dedicated to science diplomacy in the Americas peaked in 2021 and has since been in decline. Funding from African sponsors was initiated in 2019 and has since experienced a steady but shallow increase. No funders from Oceania have been reported for this study’s sample of science diplomacy publications.

**Fig. 7:**
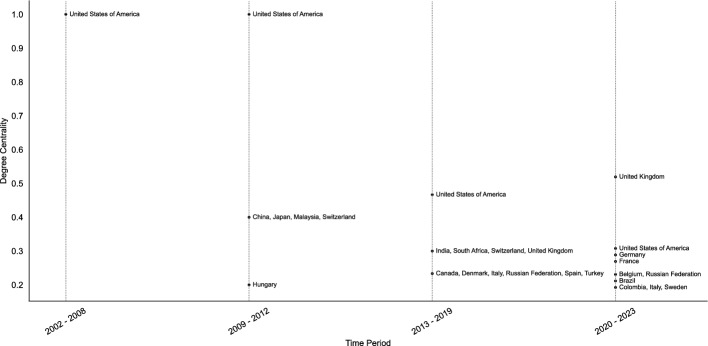
Countries with the ten highest degree centralities over time. Where several countries have the same degree centrality, more than ten countries may be listed. Countries with the same degree centrality are moreover grouped together

Figure 7, which ranks countries with the ten highest degree centralities in the collaboration network (i.e. who collaborates with whom on a publication) for the period 2002–2023, features 21 countries (see Fig. 7). These 21 countries represent four regions, with 11 representing Europe, five Asia, four the Americas and one Africa, with Oceania not having any representation. Overall, the figure demonstrates an important shift from the US as the most central actor in the science diplomacy scholarship to a gradual internationalization of collaborations starting in the period 2013–2019.

In the first period (2002–2008) covered in Fig. 7, the degree centrality of the US and the fact that no other country has a degree centrality above zero, implies that the US is the only country that produced scholarship on science diplomacy from 2002–2008. This highlights that the very limited science diplomacy scholarship that emerged during this particular time bracket (there are less than 10 science diplomacy publications for the 2002–2008 period; compare also Fig. 4 and Fig. 5) is shaped by one single country and is thus far from international.

During the period 2009–2012, a range of countries, namely China, Japan, Malaysia, Switzerland, and Hungary, have a degree centrality that is higher than 0.1, indicating that they have begun to produce collaborative scholarship on science diplomacy. At this point in time, the degree centrality of the US still equals one, which implies that it remains the most central actor in the collaboration network that all other countries collaborate with on science diplomacy publications.

Considering the period 2013–2019, it is possible to observe a more pronounced internationalization of collaborations than in the previous time bracket. While the US is clearly still the most central actor, its degree centrality has decreased from 1 to 0.5, indicating that it has ceased to be the hub that all other countries collaborate with. Instead, there are several countries in the top 10, such as India, South Africa, Switzerland, the UK, Canada, Denmark, Italy, the Russian Federation, Spain, and Turkey, that have a degree centrality above 0.2. This implies that these countries have started to collaborate with more countries than just the US.

Finally, during the period 2020–2023, the US has lost its position as the most central collaborating actor to the UK. The latter’s centrality is almost twice as high as that of the US in this period. In addition, collaborations in the science diplomacy scholarship have further diversified. In the three-year period from 2020–2023, countries like Germany, France, Belgium, the Russian Federation, Brazil, Colombia, Italy, and Sweden have begun to collaborate more widely. Moreover, countries that had a high degree centrality in the previous time bracket, such as India, South Africa, and Canada, are not represented in the top 10 for the period 2020–2023. Only the Russian Federation, Italy, and the UK are represented in the two most recent periods under examination.

**Fig. 8:**
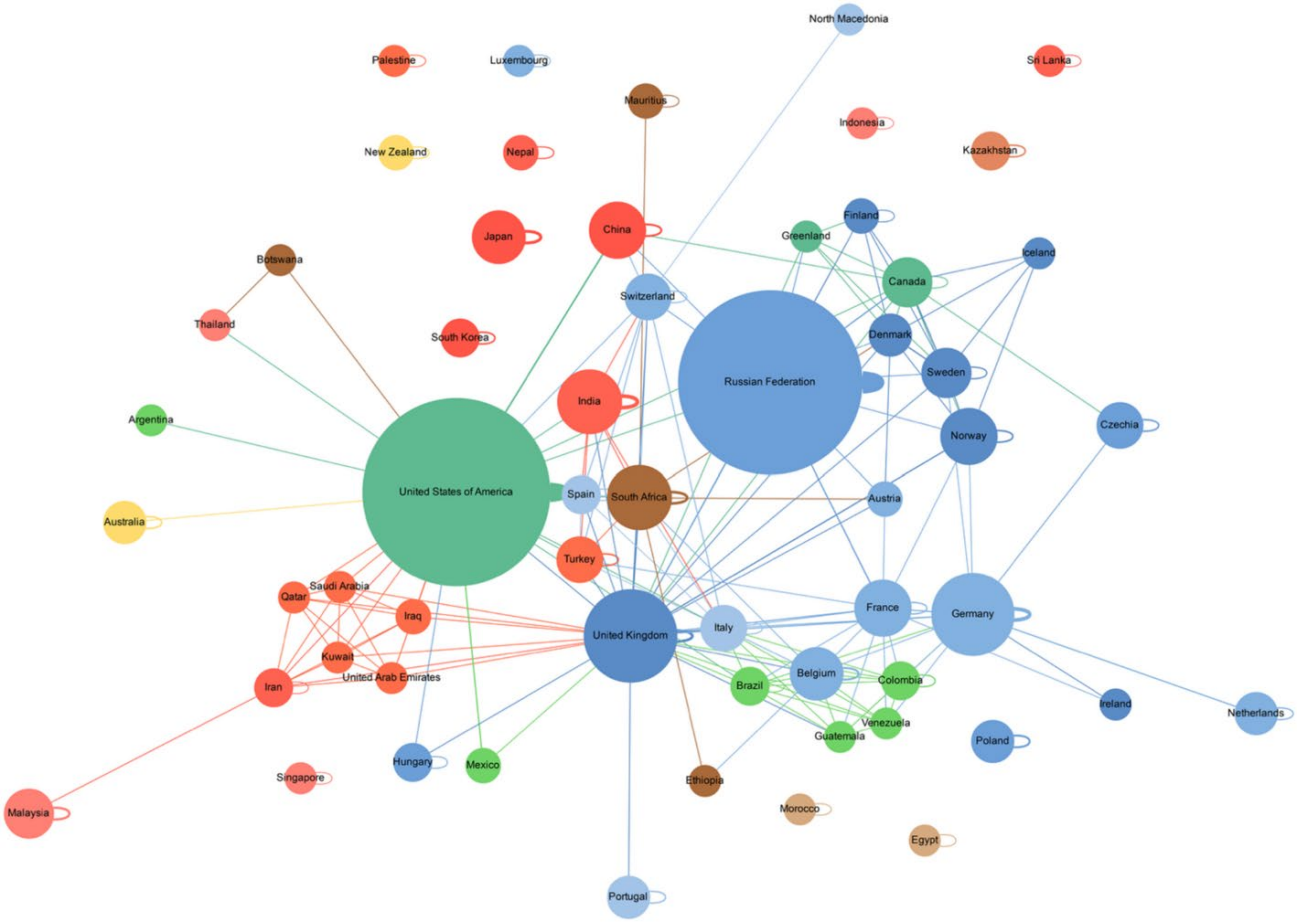
Collaboration network 2002-2023; Click this link to access an interactive version of the network based on the study’s dataset: https://brunogrisci.github.io/scidip/authornet_2002_2023.html

Considering the collaboration network that covers the complete period under examination (2002–2023; see Fig. 8), with the size of nodes being proportional to the number of papers authored by scholars from that country, it is clear that collaborative work by scholars in the science diplomacy field based in a range of countries has been on the rise.

In terms of individual countries, it is worth highlighting several observations. First, the US is very notable in the collaboration network, given its early dominance in collaborative science diplomacy research. Second, the Russian Federation has begun to rival the United States as one of the most prominent producers of science diplomacy scholarship. The loops around both country nodes (which represent self-connections) indicate that a considerable share of publications in the US and the Russian Federation result from national collaborations. Third, there is a considerable number of countries, among them New Zealand, Japan and South Korea, that do not collaborate with any other countries on science diplomacy publications.

Seen from the regional level, the collaboration network covering the complete period under examination moreover shows that Europe dominates the scholarship on science diplomacy. At the same time, Asia and Latin America have developed into noticeable hubs of the science diplomacy scholarship, although the number of contributions from these two regions are clearly below those coming from Europe.

**Fig. 9:**
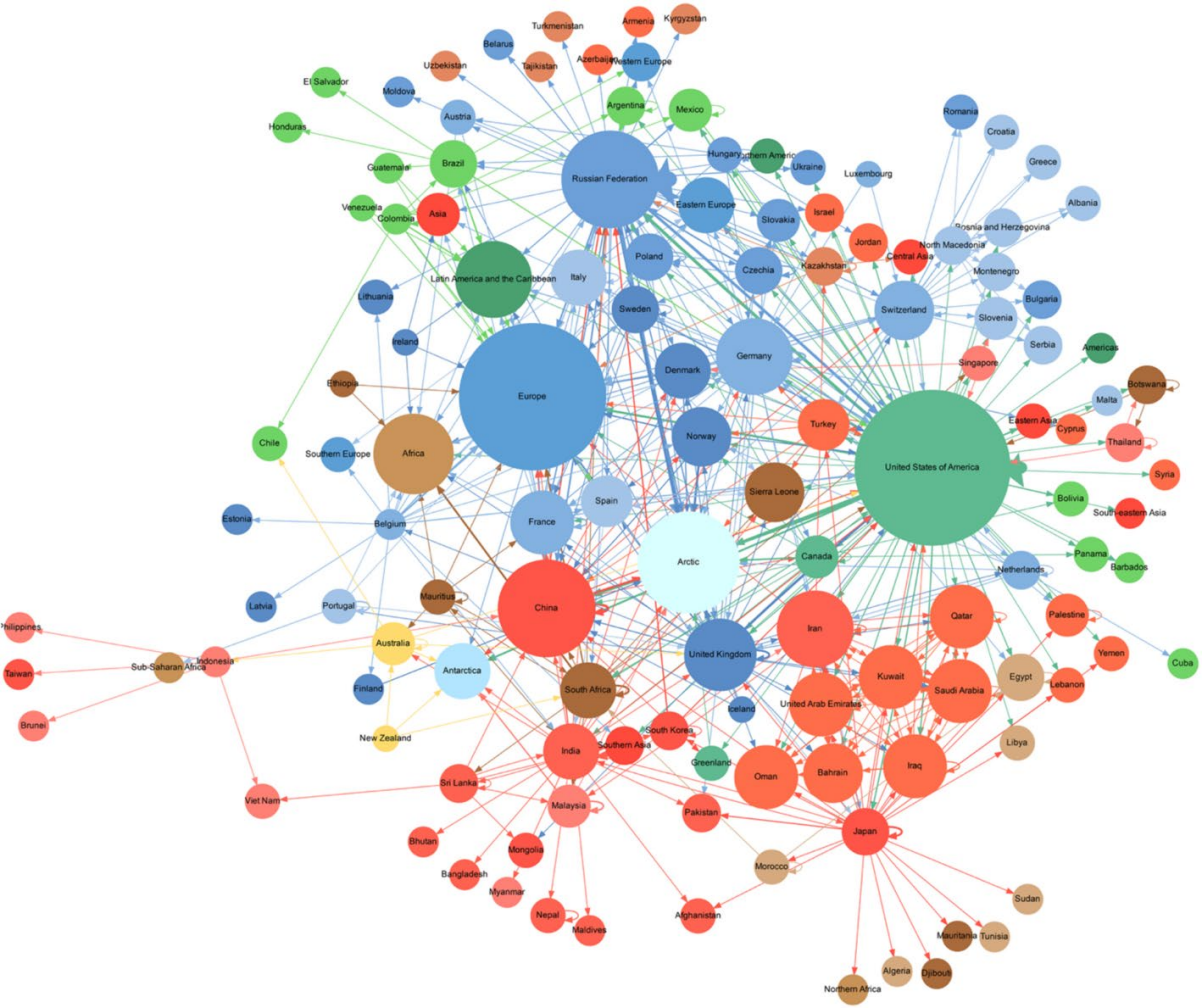
Target network 2002 -2023; Click this link to access an interactive version of the network based on the study’s dataset: https://brunogrisci.github.io/scidip/regionnet_2002_2023.html

**Fig. 11:**
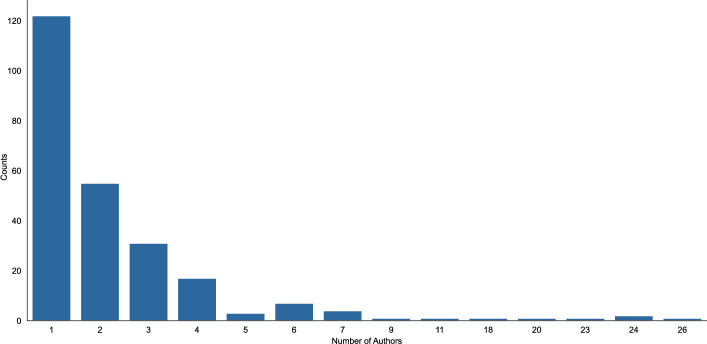
Number of authors per publication

When examining the target network (i.e. who mentions who in an abstract) in the period 2002–2023 (see Fig. 9), where node sizes are proportional to their in-degree centrality, indicating their importance in relation to the number of inward-going links and the width of arrows is proportional to the number of connections between nodes (more connections from node A to node B are represented by a thicker arrow), it is clear that the main targets of interest in the science diplomacy literature have been the US and Europe. Interest in the US has not only been high internationally, but also domestically (represented by self-connections).

In terms of regions (apart from Europe), the Arctic, Africa as well as Latin America and the Caribbean have been the most studied in this period by science diplomacy scholars. Regionally, there has been a strong internal focus among Western Asian countries (including, for example, but not limited to Kuwait, Qatar, Saudi Arabia and the United Arab Emirates).

Country wise, after the US, the Russian Federation, China, Germany, the UK, Switzerland, France, Denmark, Sweden, Norway, South Africa, and India, have all received particular attention in the literature. When looking at the science diplomacy scholarship of the Russian Federation and the US specifically, there has been a remarkable national focus. Moreover, Russian-based scholars have also had a significant interest in science diplomacy in relation to the Arctic and the US.

### Discussion and implications

The objective of this study was twofold: It set out to examine (i) how global, as opposed to national, the scholarship is in terms of authorship, funding, and outlook as well as (ii) investigating to what extent recent initiatives to globalize the field are reflected in these three dimensions.

With respect to the first research objective, the study’s results clearly demonstrate that, overall, there is a strong national focus in the science diplomacy scholarship. For instance, the collaboration and target networks show that a considerable share of the science diplomacy scholarship is national in character, with central actors like the US and the Russian Federation demonstrating a pronounced interest in studying their own science diplomacy approaches. This finding contrasts with the practitioner-driven discourse on science diplomacy. Only the scholarship’s continuous focus on global commons, such as the Arctic, Antarctica, and the Atlantic, somewhat counters the national trend in the production of knowledge on science diplomacy. Both the Arctic and Antarctica have remained popular topics of study throughout the period covered in the dataset, more so than the Atlantic.

The study’s descriptive statistics moreover show that when science diplomacy scholars collaborate internationally, they typically do so across only one national boundary, meaning that collaborations involving two countries are particularly common. Intellectual fertilization across multiple regional boundaries is similarly rare. Given the practitioner-driven discourse on science diplomacy as key to tackle global challenges and the transboundary character of these challenges, more international collaboration could have been expected. Instead, it seems that relatively closed-off national and intra-regional communities play an important role in advancing the field. There are three factors which can explain this pattern. First, the fields of study which predominantly contribute to the science diplomacy scholarship (e.g. history, political science, science and technology studies as well as international relations) are known to have authors publish alone or in small teams (Lewis et al., 2012; Thelwall & Maflahi, 2022). As a result, collaborations, including international ones, are less common in these fields than they are in others, for example in the natural sciences (Larivière et al., 2006). Second, it is challenging for science diplomacy scholars to acquire the in-depth knowledge of several idiosyncratic national science systems that is required to conduct meaningful cross-country and cross-regional science diplomacy case studies. Third, as has been outlined in the literature review, science and innovation are typically considered an important cornerstone of a nation’s knowledge economy. Policymaking in this realm is accordingly often considered a national issue, which could explain why scholars tend to focus on this level of analysis.

Concerning the study’s second research objective, the data clearly shows that knowledge production on science diplomacy has only started to become more diverse in recent years and more so when it comes to the regions that are targeted in publications, than the geographical distribution of authors. For instance, the number of science diplomacy scholars based in Asia and Africa has only markedly increased in 2023, especially if seen in proportion to the number of European-based science diplomacy authors. At the same time, the ranking chart illustrates that the US is losing importance in the more recent science diplomacy scholarship. Its decline as the major contributor to the recent science diplomacy scholarship may be attributed to a change in US science and foreign policy from 2017 onwards (Plumer & Davenport, 2019). In comparison to the affiliation of science diplomacy scholars, the focus of science diplomacy publications has become more diverse. For instance, Latin America and the Caribbean have developed into important sub-regions of study starting in 2014 and remaining in this position until 2023. This increased focus on the global South—Asia and Latin America in particular—as an object of study can be explained by the fact that several special issues and edited volumes on science diplomacy in the global South and Latin America have been published during the past few years (e.g. 2021 Frontiers Special Issue on Science Diplomacy and Sustainable Development: Perspectives from Latin America; 2021 Centaurus Special Issue on Global Perspectives on Science Diplomacy; 2023 edited volume on Science, Technology and Innovation Diplomacy and Developing Countries: Current Issues and Challenges) (Adamson & Lalli, 2021; Bonilla et al., 2021; Ittekot & Bandopadhyay, 2023). The impact of only one such initiative on the diversity of the science diplomacy scholarship can easily be demonstrated. For instance, if the chapters (n = 7) of the edited volume on “Science, Technology and Innovation Diplomacy and Developing Countries” (Ittekot & Bandopadhyay, 2023) were to be excluded from the study’s dataset, the representation of scholars based in Africa would decrease by half (from 14 to 7) for the year 2023.

The study’s data thus suggest that recent calls to globalize the science diplomacy scholarship have helped diversify the geographical focus of the science diplomacy scholarship. These initiatives have, however, failed to affect the structural barriers that prevent scholars based in the global South from contributing to the production of knowledge on science diplomacy. This is unsurprising given that many of these barriers are the result of persistent power asymmetries between “the West and the Rest” (Mahbubani, 1992). For instance, countless previous studies have shown that researchers in the global South work under severe funding constraints (Lee et al., 2023; Teferra et al., 2022). In line with this, the descriptive funding data of this study demonstrates that during the past twenty years a majority of science diplomacy publications have been funded by relatively prosperous European countries and the EU. The latter’s remarkable investments into science diplomacy research can be explained by the EU’s identity as a market and normative power, as one study on European science diplomacy suggests (López de San Román & Schunz, 2018). The study argues that the EU is keen to promote science diplomacy for two main reasons. First, external action in the science domain helps the EU to defend its interests in research and development as well as to spread its practices, policies and regulations globally (López de San Román & Schunz, 2018, p. 4). Second, external science activities allow the EU to establish cooperation with third countries based on European norms as well as to promote such norms where necessary (López de San Román & Schunz, 2018, p. 4). In keeping with the EU’s market-driven motivation to promote science diplomacy, it is likely that while Brussels might encourage collaborations between European and researchers based in other world regions, its intention is for the bulk of the allocated resources to stay within the EU. Accordingly, most of the researchers that contributed to the three EU-funded projects on science diplomacy (El-CSID, S4D4C, and InsSciDE) were affiliated with a research institution in a EU Member State (see: European Commission, 2023; S4D4C, n.d.). In conclusion, this means that as long as national funding for science diplomacy research in the global South does not increase and Western funders do not encourage more international research collaboration on science diplomacy, the global South is likely to remain at the periphery of knowledge production on science diplomacy. The funding trends clearly support this conclusion, as a recent growth in Asian science diplomacy funding has also led to an increase in science diplomacy publications by scholars based in Asia. The fact that only Southern emerging powers with the necessary funding as well as relatively strong science and technology sectors, such as South Africa, India, and China, are fairly well-embedded in the collaboration network further highlights the pivotal role that resources play in the global production of knowledge, even in research fields like science diplomacy that are less resource intensive.

From these findings follow two important implications for the science diplomacy field and community. First, due to the comparatively small size of the science diplomacy scholarship, it is possible to observe how even small changes in publication patterns impact the overall diversity of the science diplomacy scholarship. For example, as described above, the exclusion of only one edited volume from the dataset has a noticeable and negative impact on the diversity of the 2023 authorship patterns. Conversely, this means that small-scale and resource-efficient collaborations like initiating an edited volume with participation from scholars based in different world regions can positively impact the internationalization of science diplomacy research. Second, the study’s findings indicate that scholars based in the global South will only be able to participate in science diplomacy knowledge production more actively, if and when their funding situation improves. As the case of Europe and the EU illustrates, an increase in funding often translates into more international collaborations, gaining a central role within a research community, and having the ability to shape the global discourse on a given research topic. If countries from the global South had more resources available, they could build up a similar collaborative structure in the field of science diplomacy as Europe did over the past few years. This would require national governments in the global South to provide funding for science diplomacy research. While this would also stand to benefit Southern countries competitive advantage on the international stage in the long run, in practice, it may prove difficult. Instead, it might be more practicable to improve access to international funding for science diplomacy scholars based in the global South. On the one hand, this would require (predominantly Western) funders to create more opportunities for international collaboration in the science diplomacy field, for instance by bringing researchers together through sponsored conferences, workshops and fellowship programmes. On the other hand, and as has been argued elsewhere (Polejack et al., 2022), it would require each and every science diplomacy scholar to make reflexivity a common and meaningful research practice. This would help nudge scholars to more deeply reflect on their collaboration (e.g. How can a collaboration be set up in an equitable manner?) and referencing practices (e.g. How many scholars based in the global South were cited in a given piece of work?). As mentioned in the article’s introduction, thinking about and tackling inequalities in the science diplomacy scholarship is not an end in itself, although it has considerable merit from an equity in science perspective. Rather, such inequalities could have broader implications, as it has repeatedly been argued that science diplomacy can directly advance a nation’s and region’s interests, thus providing those with the necessary capacities and knowledge to engage in science diplomacy with a political and economic advantage.

## Conclusion and limitations

This study is the first to provide a bibliometric mapping of the science diplomacy scholarship along geographical criteria. In line with previous research (Feld & Kreimer, 2019; Schubert & Sooryamoorthy, 2010), its results show that the science diplomacy research field has distinct North–South and center-periphery divisions. As has been shown elsewhere (Rungius & Flink, 2020; Turchetti et al., 2020), the study also demonstrates that the science diplomacy concept originated in the US from where it spread to other world regions. Future research could investigate whether the concept of science diplomacy has been adapted and reinterpreted during this transfer process, to better align with other national or regional concerns and political priorities. Based on the result of the bibliometric analysis, the study has moreover outlined two concrete proposals as to how the field of science diplomacy can live up to its international reputation and recent calls to globalize science diplomacy research. Building on these proposals, additional, qualitative research could help identify best practices for more equitable and diverse research collaborations in the field of science diplomacy.

In addition, this study provides a proof of concept for an LLM-enhanced bibliometric analysis. In line with previous research that has explored the potential of LLMs in automating systematic literature reviews (De Silva et al., 2024; Gargari et al., 2024; Guo et al., 2024), this study demonstrates that LLMs like GPT-4 make for a potent research assistant in bibliometric studies by accelerating and scaling the data collection and refinement process beyond human capabilities. Future research should further extend the methodological approach suggested in this study by testing how well GPT-4 performs in identifying whether the main text of a publication, not just its abstract, is relevant considering some predefined conceptual criteria. Moreover, additional studies could compare how well GPT-4 performs in comparison with other state-of-the-art models like Claude (Anthropic, 2024) or free-to-use models like Llama (Meta, n.d.). The dataset created for this study can be used as a starting point for such bench-marking excercises as well as future experiments with LLMs.

Finally, it is important to underline that this study comes with five methods-related limitations, which should directly inform future research. First, Scopus data only covers peer-reviewed publications. While practitioners have contributed some of these publications, a large share of the practitioner-driven literature on science diplomacy is not indexed in Scopus. For a field like science diplomacy, that is considerably shaped by practitioner-driven contributions, a study like the present one may thus only tell part of the story. This limitation can be tackled in future research, for instance through a bibliometric study which traces how and to what extent practitioner-driven contributions like white papers and opinion pieces are cited in the scholarly literature. This would provide the science diplomacy community with an indication as to how important gray literature has been and continues to be for the field’s overall development. Using Scopus as a bibliometric database comes with a second limitation, as Scopus data only indicates where an author is based, not whether this is also where a scholar is originally from. As a result, this study may, for example, have labeled someone that is originally from Latin America, but is currently associated with a research institution in Europe, as a “European” scholar. Third, as was highlighted in the method section, this study exclusively focused on publications in English, which biases its results in favor of scholarship originating from Europe and North America. Future studies may therefore want to use bibliometric databases which have a broader linguistic coverage, such as Ulrichsweb or OpenAlex. However, such an undertaking comes with considerable challenges, as it would require an intimate knowledge of how key terms (e.g. science diplomacy) are used in different languages. Fourth, the data selection for this study was done on the basis of a restrictive set of search terms. Future bibliometric studies could use a more inclusive set of search terms, thus increasing the number of science diplomacy-related publications for analysis. Finally, and as has already been indicated in the methods section, aggregating data at the country, sub-regional, and regional level comes with certain challenges. The decision to use contemporary country names instead of historical ones, for example, is likely to have artificially inflated the centrality of Russia in the target network, as all publications referring to the Soviet Union (n = 40) were labeled as Russia-related. Future research may therefore want to use both historical and contemporary country names.

Yet, even with these limitations in mind, this contribution is significant in two respects. First, from a methodological point of view, it contributes a useful LLM-enhanced approach to data collection that can be used for other bibliometric studies. Second, from a science diplomacy perspective, it provides policymakers, funders, and the science (diplomacy) community with an overview of how global the field of science diplomacy has become to date. In doing so, this study highlights where future funding and collaborative initiatives could focus their attention, to catalyze more diverse, global, and equitable research at the intersection of international relations and science.

## Figures and Tables

**Fig. 10:**
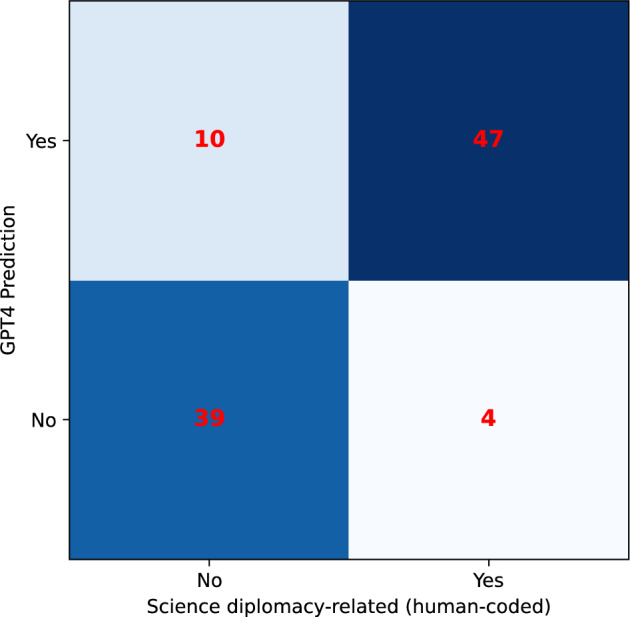
Confusion matrix

## Data Availability

The data and code that support the findings of this study are openly available in GitHub: https://github.com/BrunoGrisci/science-diplomacy-global-research.

## Appendix

See Figs. 10 and 11

## Code availability

The data and code that support the findings of this study are openly available in GitHub: https://github.com/BrunoGrisci/science-diplomacy-global-research.
